# High triglyceride-glucose index is associated with subclinical cerebral small vessel disease in a healthy population: a cross-sectional study

**DOI:** 10.1186/s12933-020-01031-6

**Published:** 2020-05-06

**Authors:** Ki-Woong Nam, Hyung-Min Kwon, Han-Yeong Jeong, Jin-Ho Park, Hyuktae Kwon, Su-Min Jeong

**Affiliations:** 1 Department of Neurology, Seoul National University College of Medicine and Seoul National University Hospital, Seoul, South Korea; 2 Department of Neurology, Seoul National University College of Medicine and Seoul Metropolitan Government-Seoul National University Boramae Medical Center, 20 Boramae-ro 5-gil, Dongjak-Gu, Seoul, 07061 South Korea; 3 Department of Family Medicine, Seoul National University College of Medicine and Seoul National University Hospital, 101 Daehak-ro, Jongno-Gu, Seoul, 03080 South Korea; 4 Department of Family Medicine, Seoul National University College of Medicine and Seoul Metropolitan Government-Seoul National University Boramae Medical Center, Seoul, South Korea

**Keywords:** Triglyceride, Glucose, Insulin resistance, Leukoaraiosis, Lacunes

## Abstract

**Background:**

The triglyceride-glucose (TyG) index is a marker of insulin resistance (IR) and has been associated with various metabolic syndromes, cardiovascular diseases, and cerebrovascular diseases. However, limited information is available regarding its association with subclinical cerebral small vessel disease (cSVD). In this study, we evaluated the relationship between the TyG index and cSVD, including silent brain infarcts (SBIs) and white matter hyperintensity (WMH).

**Methods:**

We assessed health check-up participants aged 40–79 years from 2006 to 2013. The TyG index was calculated using the log scale of fasting triglyceride (mg/dL) × fasting glucose (mg/dL)/2. The Homeostatic Model Assessment for Insulin Resistance (HOMA-IR) was also calculated. This was compared with two insulin surrogates and cSVD as another IR indicator and compared the association between two insulin surrogates and cSVD. SBI was measured for both prevalence and burden. The WMH volume was quantitatively rated using a computer-assisted semi-automated technique.

**Results:**

A total of 2615 participants were evaluated (median age: 56 years, male sex: 53%). In the multivariable logistic regression analysis, the TyG index was seen to be associated with SBI prevalence (adjusted odds ratio: 1.39; 95% confidence interval [CI] = 1.06–1.81). Further quantitative analyses showed a positive dose–response relationship between the TyG index and SBI burden (*P* for trend = 0.006). In multivariable linear regression analysis, the TyG index was also found to be related to the volume of WMH (β = 0.084; 95% CI = 0.013 to 0.154). Additionally, the TyG index showed a similar or slightly stronger association with the prevalence of SBI and the volume of WMH than did HOMA-IR.

**Conclusions:**

A high TyG index was associated with a higher prevalence and burden of cSVD in a neurologically healthy population. This marker of IR could be a convenient and useful predictor of cSVD.

## Background

Insulin resistance (IR) is a pathological condition that results from poor insulin sensitivity in the peripheral tissues [1]. IR increases the risk of metabolic syndromes, cardiovascular diseases, and cerebrovascular diseases. Therefore, it is necessary to accurately measure IR in clinical practice [1–5]. Although hyperinsulinemia-euglycemic clamps are considered to be the gold standard in quantifying insulin sensitivity, they are expensive, time-consuming, and labor intensive [6, 7]. As an alternative, the Homeostatic Model Assessment for Insulin Resistance (HOMA-IR) has been proposed. However, the HOMA-IR requires insulin levels that are not usually measured in clinical practice [8].

Patients with IR often show lipid metabolism disorders as well as glucose metabolism disorders. Excessive lipolysis causes circulating free fatty acids to increase, along with increase in the synthesis of hepatic triglyceride (TG) and very low-density lipoprotein [4, 9, 10]. Therefore, the following dyslipidemia pattern is typically seen in IR: high TG, low high-density lipoprotein (HDL), and high small-sized low-density lipoprotein (LDL) particles [1, 4, 11]. Based on this theoretical background, the triglyceride-glucose (TyG) index using fasting TG and fasting glucose was suggested as a convenient IR marker, which showed a good correlation with hyperinsulinemia-euglycemic clamps and the HOMA-IR [12]. Like other IR markers, the TyG index was also found to be associated with metabolic syndromes, cardiovascular diseases, cerebrovascular diseases, arterial stiffness, and carotid atherosclerosis [13–21]. Besides, some studies have also shown a stronger association with these conditions and the TyG index than with HOMA-IR [11, 19]. However, the relationship with subclinical cerebral small vessel disease (cSVD) is still unknown.

cSVD is a subclinical pathology that includes various pathological subtypes such as white matter hyperintensity (WMH) and silent brain infarcts (SBIs) [22]. Since cSVD increases the risk of ischemic stroke or vascular dementia [23, 24], early identification of the high-risk group is necessary. As a risk factor, many studies that have focused on the relationship between IR and cSVD have used the HOMA-IR [25, 26]. However, if a relationship can be found between the TyG index which can be obtained with a simple test and cSVD, the high-risk group will be able to be identified more easily. In this study, we examined the association between the TyG index and cSVD in a neurologically healthy population. Additionally, the association between the different subtypes of cSVD and the TyG index were examined to investigate whether IR acts as a common pathological mechanism of cSVD development or has different impacts depending on the cSVD subtypes.

## Methods

### Patients and population

This retrospective cross-sectional study was approved by the Institutional Review Board (IRB number: H-1502-026-647) of Seoul National University Hospital. The requirement of written informed consent from participants was waived by the IRB for this study due to the retrospective design and because only de-identified and anonymized participant information was used. All experiments were performed in accordance with the Declaration of Helsinki and all relevant guidelines and regulations. Any data not published within the article are available from the corresponding author upon reasonable request.

Previously, we constructed a registry of consecutive health check-ups that were conducted at the Seoul National University Hospital Health Promotion Center between 2006 and December 2013 [25, 27, 28]. The health check-up registry includes medical history information that was self-reported by the participants. Additionally, laboratory results and radiological examination findings, which were conducted according to our center’s protocol were included in the final registry. As a part of this registry, participants aged 40–79 years were included (n = 3132). Among them, 64 participants with a history of stroke or a severe neurological deficit were excluded. Furthermore, we excluded participants using (1) lipid-lowering agents (n = 258), (2) glucose-lowering agents (n = 154), and those (3) missing data needed to calculate TyG index (n = 41). Finally, a total of 2615 neurologically healthy participants were included in the final cross-sectional analyses.

### Demographic, clinical, and laboratory assessment

We evaluated the demographic factors, clinical factors, and vascular risk factors that were measured in the routine health check-ups. Age, sex, body mass index (BMI), systolic and diastolic blood pressure (BP), and use of medications (i.e., antihypertensives, antiplatelet agents) were assessed [27, 29]. Laboratory examinations were carried out after 12 h of overnight fasting, and included glucose profiles, insulin levels, lipid profiles, white blood cell (WBC) counts, and high-sensitivity C-reactive protein (hs-CRP) levels [27, 29].

The TyG index was calculated using the log scale of [fasting TG (mg/dL) × fasting glucose (mg/dL)/2], used in previous studies [8, 19]. To compare the association between the peripheral IR (TyG index) and traditional hepatic IR (HOMA-IR) methods with cSVD, we also calculated the HOMA-IR using the following equation: glucose (mg/dL) × insulin (µU/mL)/405 [25].

### Radiological assessment

All participants underwent brain magnetic resonance imaging (MRI) using 1.5-T MR scanners (Signa [GE Healthcare, Milwaukee, WI, USA] or Magnetom SONATA [Seimens, Munich, Germany]). The detailed acquisitions of MRI were as follows: T1-weighted images, repetition time (TR)/echo time (TE) = 500/11 ms; T2-weighted images, TR/TE = 5000/127 ms; T2 fluid-attenuated inversion recovery images, TR/TE = 8800/127 ms; and T2-gradient echo images, TR/TE = 57/20 ms. The basic slice thickness of the brain was 5 mm. SBI was defined as an asymptomatic, well-defined lesion, more than 3 mm in size with the same signal characteristics as cerebrospinal fluid on T1 and T2 images [22, 29]. We rated the burden of SBI as absent, single, or multiple, based on the number of SBI lesions present [29]. WMH volume was quantitatively rated using a computer-assisted semi-automated technique (MIPAV, version 7.3.0., National Institutes of Health, Bethesda, MD) [27]. All radiological measurements were rated by two experienced neurologists (K.W.-N. and H.-Y.J.), who were blinded to the other clinical data. Any disagreements were resolved through a discussion with a third rater (H.-M.K.).

### Statistical analysis

All statistical analyses were conducted using SPSS version 21.0 (IBM SPSS, Chicago, IL, USA). Continuous variables with a normal distribution are presented as a mean ± standard deviation, and those with non-normal distribution as a median [interquartile range]. Continuous variables with skewed data were transformed into a log scale, except for WMH volume, which was transformed into a square root scale due to the existence of many zero values. Since the prevalence of SBI is a binary event outcome, we conducted univariate analyses using either the Student’s t-test or the Mann–Whitney *U*-test for continuous variables and the Chi squared or Fisher’s exact tests for categorical variables. In the case of WMH volume, because the outcome itself is a continuous variable, we performed the univariate analysis using a simple linear regression analysis. Then, statistically significant (*P* < 0.05 in the univariate analysis) and clinically important (sex and BMI) variables were introduced in the multivariable analyses. Since the TyG index is itself composed of glucose and TG values, the glucose and lipid profiles were not simultaneously included in the multivariable analysis as confounders.

To assess the dose–response relationship between insulin sensitivity surrogates and SBI burden, we compared the median TyG index and HOMA-IR among participants with different burdens of SBI. In this analysis, we used the Jonckheere–Terpstra test to obtain tendencies (*P* for trend). The relationship between the TyG index and vascular risk factors were also examined using simple linear regression analyses. This was done to gain insights into mechanisms that show an association between the TyG index and cSVD. A *P*-value of < 0.05 was considered statistically significant.

## Results

Our study included a total of 2615 neurologically healthy participants (mean age: 57 years, male sex: 53%). The mean volume of WMH was 2.53 ± 5.78 mL, and SBIs were found in 209 (8%) participants. The mean value of the TyG index was 8.47 ± 0.57. Other detailed baseline characteristics are presented in Additional file 1: Table S1.

**Fig. 1:**
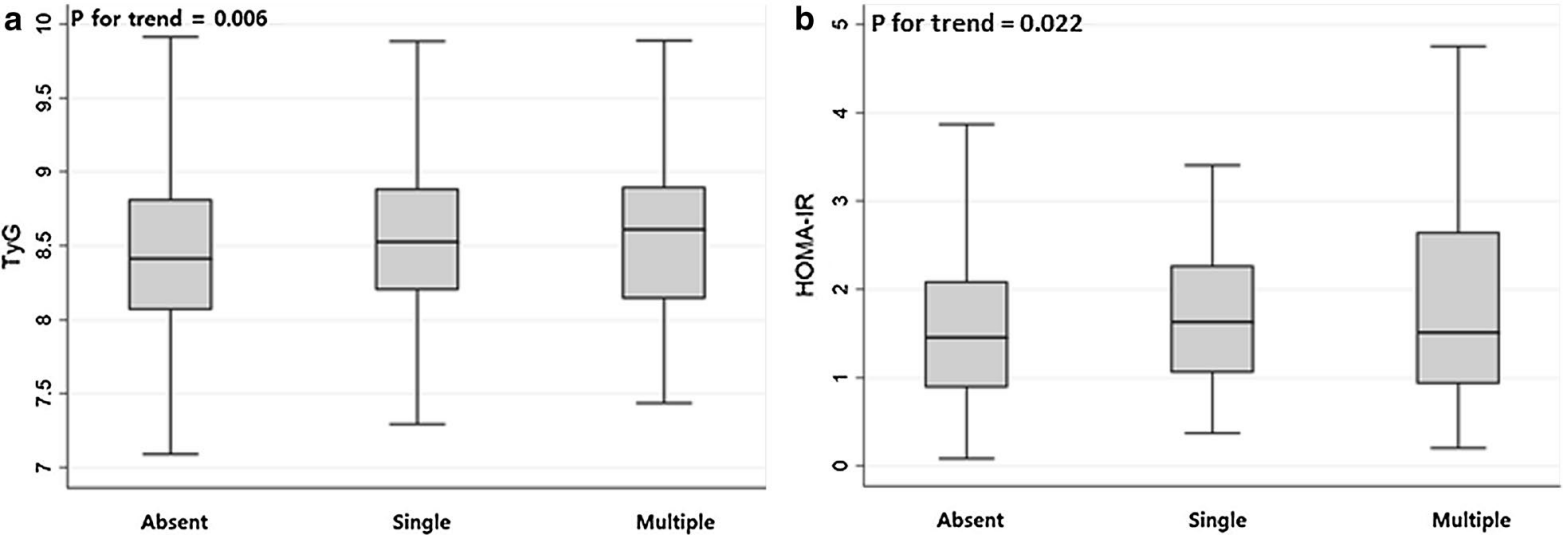
Distributions of the insulin sensitivity surrogates according to the burden of silent brain infarcts (SBIs). Patients with multiple SBI lesions had a higher TyG index (*P* for trend = 0.006) and HOMA-IR (*P* for trend = 0.022) than those with no SBI lesions or a single lesion in a dose–response manner

In the univariate analysis, it was found that the presence of SBIs was related to age, use of antihypertensives and antiplatelet agents, systolic and diastolic BP, level of HbA1c, fasting glucose, HOMA-IR, total/LDL/HDL cholesterol, TG, hs-CRP, and the TyG index (Table 1). In the multivariable logistic regression analyses, the TyG index was found to be significantly associated with SBI (adjusted odds ratio [aOR] = 1.39; 95% confidence interval [CI] = 1.06–1.81) after adjusting for confounders. Age (aOR = 1.09; 95% CI = 1.07–1.11) and use of antihypertensives (aOR = 1.41; 95% CI = 1.00–1.97) were also related to SBI (Table 2). Compared to HOMA-IR, the TyG index had a larger aOR value and a smaller *P*-value, indicating a stronger association with the prevalence of SBI (Table 3). In the evaluation of the relationship between IR surrogates and the burden of SBI, patients with multiple SBI lesions had a higher TyG index (*P* for trend = 0.006) and HOMA-IR (*P* for trend = 0.022) as compared to the others, showing a dose–response manner (Fig. 1).

**Table 1:** Baseline characteristics of SBI (+) and SBI (−) groups

	No SBI (n = 2406)	SBI (n = 209)	*P*-value
Age, years [IQR]	56 [50–62]	63 [57–68]	< 0.001
Sex, male, n (%)	1273 (53)	113 (54)	0.748
Body mass index, kg/m^2^ [IQR]	23.89 [22.09–25.77]	23.97 [21.99–26.09]	0.504
Use of antihypertensives, n (%)	415 (17)	59 (28)	< 0.001
Use of antiplatelet agents, n (%)	185 (8)	25 (12)	0.029
Systolic BP, mmHg [IQR]	125 [115–135]	131 [120–142]	< 0.001
Diastolic BP, mmHg [IQR]	75 [69–83]	78 [70–87]	< 0.001
Hemoglobin A1c, % [IQR]	5.7 [5.5–5.9]	5.8 [5.5–6.2]	< 0.001
Fasting glucose, mg/dL [IQR]	91 [84–99]	92 [85–105]	0.015
Insulin, µU/mL [IQR]^*^	6.5 [4.1–8.9]	6.9 [4.6–9.7]	0.057
HOMA-IR [IQR]^*^	1.46 [0.89–2.09]	1.58 [1.01–2.34]	0.022
Total cholesterol, mg/dL [SD]	203 ± 36	197 ± 35	0.030
LDL cholesterol, mg/dL [IQR]	129 [107–151]	121 [97–150]	0.041
HDL cholesterol, mg/dL [IQR]	54 [45–64]	51 [44–61]	0.043
Triglyceride, mg/dL [IQR]	98 [72–140]	104 [77–145]	0.041
WBC counts, × 10^3^/μL [IQR]	5.25 [4.34–6.30]	5.47 [4.51–6.55]	0.051
hs-CRP, mg/dL [IQR]	0.04 [0.01–0.14]	0.08 [0.01–0.18]	0.024
TyG index [IQR]			0.007

SBI: silent brain infarct; BP: blood pressure; HOMA-IR: Homeostatic Model Assessment for Insulin Resistance; LDL: low-density lipoprotein; HDL: high-density lipoprotein; WBC: white blood cell; hs-CRP: high-sensitivity C-reactive protein; TyG index: triglyceride-glucose index

^*^These variables were measured in 2051 participants

**Table 2:** Multivariable logistic regression analyses between possible predictors and silent brain infarct

	Unadjusted OR (95% CI)	*P*-value	Adjusted OR^†^ (95% CI)	*P*-value^†^
Age, years	1.09 [1.07–1.11]	< 0.001	1.09 [1.07–1.11]	< 0.001
Sex, male	1.05 [0.79–1.39]	0.748	0.98 [0.73–1.31]	0.872
Body mass index, kg/m^2^	1.02 [0.98–1.07]	0.305	1.01 [0.96–1.06]	0.835
Use of antihypertensives	1.89 [1.37–2.60]	< 0.001	1.41 [1.00–1.97]	0.048
Use of antiplatelet agents	1.63 [1.05–2.54]	0.031	1.01 [0.63–1.62]	0.959
hs-CRP, mg/dL^*^	1.13 [1.03–1.24]	0.008	1.07 [0.97–1.18]	0.170
TyG index	1.44 [1.13–1.82]	0.003	1.39 [1.06–1.81]	0.017

hs-CRP: high-sensitivity C-reactive protein; TyG index: triglyceride-glucose index

^*^These variables were transformed into a log scale

^†^Adjusted with *P* < 0.05 in univariate analysis (age, use of antihypertensives, use of antiplatelet agents, hs-CRP, and TyG index) and sex and BMI

**Table 3:** Multivariable analyses between possible predictors and silent brain infarct/white matter hyperintensity volume using HOMA-IR instead of TyG index

	Silent brain infarct^†^		White matter hyperintensity^‡^	
	Adjusted OR (95% CI)	*P*-value	β (95% CI)	*P*-value
Age	1.09 [1.07–1.11]	< 0.001	0.054 (0.049 to 0.059)	< 0.001
Sex, male	0.87 [0.63–1.22]	0.426	0.023 (− 0.063 to 0.109)	0.601
Body mass index	0.99 [0.94–1.05]	0.816	− 0.012 (− 0.027 to 0.003)	0.124
Use of antihypertensives	1.55 [1.07–2.25]	0.021	0.275 (0.165 to 0.384)	< 0.001
Use of antiplatelet agents	0.93 [0.51–1.68]	0.801	− 0.060 (− 0.227 to 0.108)	0.484
hs-CRP^*^	1.03 [0.93–1.15]	0.553	–	–
WBC counts	–	–	0.029 (0.003 to 0.055)	0.031
HOMA-IR^*^	1.32 [1.02–1.71]	0.036	0.075 (0.013 to 0.137)	0.018

HOMA-IR: Homeostatic Model Assessment for Insulin Resistance; hs-CRP: high-sensitivity C-reactive protein; WBC: white blood cell

^*^These variables were transformed into a log scale

^†^This analysis used a multivariable logistic regression analysis adjusted with *P* < 0.05 in univariate analysis (age, use of antihypertensives, use of antiplatelet agents, hs-CRP, and HOMA-IR) and sex and BMI

^‡^This analysis used a multivariable linear regression analysis adjusted with *P* < 0.05 in univariate analysis (age, use of antihypertensives, use of antiplatelet agents, White blood cell counts, and HOMA-IR) and sex and BMI

The TyG index was also seen to have a close relationship with WMH volume (β = 0.084; 95% CI = 0.013 to 0.154) in the multivariable linear regression analysis. Additionally, age, use of antihypertensives, and WBC counts were positively associated with the WMH volume (Table 4). The TyG index showed similar β and *P*-values as that of HOMA-IR in relation to WMH volume (Table 3).

**Table 4:** Simple and multiple linear regression analyses between possible predictors and white matter hyperintensity volume^†^

	Univariate analysis		Multivariable analysis	
	β (95% CI)	*P*-value	β (95% CI)	*P*-value
Age, years	0.056 (0.051 to 0.060)	< 0.001	0.054 (0.050 to 0.059)	< 0.001
Sex, male	0.025 (− 0.057 to 0.107)	0.555	− 0.012 (− 0.089 to 0.065)	0.756
Body mass index, kg/m^2^	− 0.001 (− 0.014 to 0.013)	0.935	− 0.008 (− 0.021 to 0.005)	0.217
Use of antihypertensives	0.384 (0.278 to 0.489)	< 0.001	0.168 (0.069 to 0.267)	0.001
Use of antiplatelet agents	0.305 (0.155 to 0.455)	< 0.001	− 0.019 (− 0.158 to 0.120)	0.785
Systolic blood pressure, mmHg	0.010 (0.008 to 0.013)	< 0.001	–	–
Diastolic blood pressure, mmHg	0.008 (0.005 to 0.012)	< 0.001	–	–
Hemoglobin A1c, %^*^	1.022 (0.614 to 1.431)	< 0.001	–	–
Fasting glucose, mg/dL^*^	0.648 (0.409 to 0.886)	< 0.001	–	–
Insulin, µU/mL^*^	0.086 (0.017 to 0.154)	0.014	–	–
HOMA-IR^*^	0.104 (0.041 to 0.166)	0.001	–	–
Total cholesterol, mg/dL	− 0.001 (− 0.002 to 0.000)	0.206	–	–
LDL cholesterol, mg/dL	− 0.002 (− 0.003 to 0.000)	0.026	–	–
HDL cholesterol, mg/dL	0.000 (− 0.003 to 0.003)	0.980	–	–
Triglyceride, mg/dL^*^	0.088 (0.005 to 0.171)	0.037	–	–
White blood cell counts, × 10^3^/μL	0.051 (0.026 to 0.075)	< 0.001	0.040 (0.017 to 0.064)	0.001
hs-CRP, mg/dL^*^	0.012 (− 0.015 to 0.040)	0.378	–	–
TyG index	0.126 (0.054 to 0.198)	0.001	0.084 (0.013 to 0.154)	0.020

HOMA-IR: Homeostatic Model Assessment for Insulin Resistance; LDL: low-density lipoprotein; HDL: high-density lipoprotein; hs-CRP: high-sensitivity C-reactive protein; TyG index: triglyceride-glucose index

^*^These variables were transformed into a log scale

^†^This variable was transformed into a square root scale

^‡^Adjusted with *P* < 0.05 in univariate analysis (age, use of antihypertensives, use of antiplatelet agents, White blood cell counts, and TyG index) and sex and BMI

The TyG index showed an association with male sex, BMI, use of antihypertensives, systolic/diastolic BP, levels of HbA1c, fasting glucose, insulin, HOMA-IR, total/LDL/HDL cholesterol, TG, hs-CRP, and WBC count (Table 5).

**Table 5:** Univariate linear regression analysis between triglyceride-glucose index (TyG) index and risk factors

	β (95% CI)	*P*-value
Age, years	0.001 (− 0.001 to 0.004)	0.333
Sex, male	0.224 (0.181 to 0.266)	< 0.001
Body mass index, kg/m^2^	0.058 (0.051 to 0.064)	< 0.001
Use of antihypertensives	0.134 (0.077 to 0.190)	< 0.001
Use of antiplatelet agents	0.064 (− 0.016 to 0.143)	0.119
Systolic blood pressure, mmHg	0.009 (0.007 to 0.010)	< 0.001
Diastolic blood pressure, mmHg	0.012 (0.010 to 0.014)	< 0.001
Hemoglobin A1c, %^*^	2.004 (1.799 to 2.208)	< 0.001
Fasting glucose, mg/dL^*^	1.833 (1.727 to 1.939)	< 0.001
Insulin, µU/mL^*^	0.358 (0.324 to 0.391)	< 0.001
HOMA-IR^*^	0.396 (0.367 to 0.426)	< 0.001
Total cholesterol, mg/dL	0.003 (0.002 to 0.003)	< 0.001
LDL cholesterol, mg/dL	0.001 (0.000 to 0.002)	0.020
HDL cholesterol, mg/dL	− 0.017 (− 0.019 to − 0.016)	< 0.001
Triglyceride, mg/dL^*^	1.100 (1.087 to 1.112)	< 0.001
White blood cell counts, × 10^3^/μL	0.095 (0.083 to 0.108)	< 0.001
hs-CRP, mg/dL^*^	0.047 (0.032 to 0.061)	< 0.001

HOMA-IR: Homeostatic Model Assessment for Insulin Resistance; LDL: low-density lipoprotein; HDL: high-density lipoprotein; hs-CRP: high-sensitivity C-reactive protein

^*^These variables were transformed into a log scale

## Discussion

In this study, we found that a high TyG index value was associated with cSVD in a neurologically healthy population. This positive association was consistent in the analyses of both WMH volume and SBI, and even showed dose–response relationships. Thus, our findings may indicate that IR could be involved in a common mechanism of two different pathologies.

It is challenging to explain the exact mechanism behind the close association between the TyG index and cSVD. However, we can suggest several possible explanations. First, hyperplasia and hypertrophy of smooth muscle cells in the arterial walls could play a role. Insulin can cause lipohyalinosis by enhancing sympathetic activity or as a growth factor [9, 11, 16, 18]. Lipohyalinosis causes diffuse cerebral hypoperfusion or blocks small perforating arterioles causing cSVD to develop [30]. Second, endothelial dysfunction should be considered. In patients with IR, subclinical inflammation and increased oxidative stress are often observed [9, 16–18, 20, 21, 31]. A strong association between the TyG index and the WBC count/hs-CRP level was also observed in the current study (Table 5). Once the vascular endothelium is impaired by inflammation, blood contents leak into the perivascular spaces, and the solutes cannot be cleared through the blood vessel wall, resulting in cSVD [30, 32]. Third, atherosclerosis could be the link between the TyG index and cSVD. In previous studies, IR was associated with the development of atherosclerosis (i.e., atherogenesis), plaque progression, and rupture [10, 16, 18, 21, 33]. Although the relationship between the TyG index and atherosclerosis was not confirmed in this study, cSVD may develop with diffuse hypoperfusion and microembolism caused by atherosclerotic lesions [30]. Last, the close association between the TyG index and cSVD could result from other concomitant metabolic syndromes. Patients with IR often have other comorbidities such as hypertension, diabetes, obesity, and low HDL cholesterol levels, as shown in Table 5 [1, 18, 19, 34]. Since these accompanying conditions are all risk factors for cSVD, the TyG index may reflect a combination of their effects.

Interestingly, in this study, the TyG index showed a similar or slightly stronger association with the prevalence of SBI and the volume of WMH than HOMA-IR. This finding was also observed in previous studies on cardiovascular and cerebrovascular diseases [11, 19], and might be because the TyG index and HOMA-IR reflect different aspects of IR [17, 33–35]. HOMA-IR represents hepatic IR, reflecting the ability of basal insulin to suppress hepatic glucose production [34, 35]. On the other hand, increased circulating TG interferes with normal glucose metabolism in muscles, so the TyG index mainly reflects to insulin sensitivity in the peripheral tissues (i.e., peripheral IR) [34, 35]. It is not clear how these differences lead to differences in the intensity of the association with cSVD. Previous studies have purported that this is because the TyG index has a stronger association with various bad metabolic status than HOMA-IR [34]. However, such differences do not appear to be large enough to account for the differences observed in either the current or previous studies. Further studies are needed to clarify this mechanism.

In previous studies, researchers have claimed that lowering IR could reduce the risk of subsequent strokes [1]. Researchers have also found that hepatic IR has bidirectional characteristics in its development. Contrastingly, peripheral IR progresses in a single direction from the abnormal TG/free fatty acid to reduce insulin sensitivity [35]. These findings indicate the possibility of reducing peripheral IR by treating preceded dyslipidemia [36]. Although our study has limitations as a cross-sectional study, it showed a clear relationship between the TyG index, a marker of peripheral IR, and cSVD. In the future, a study should be conducted that uses the TyG index as a therapeutic target and sees the effect on the development and progression of cSVD. Such a study may be able to provide insights that can help in the primary prevention of cerebrovascular diseases at subclinical levels.

There are several limitations to the current study. First, this study is a retrospective observational study that includes a large number of healthy participants. In selecting the study participants, we excluded people taking glucose- and lipid-lowering agents, which may cause selection bias. However, these medications can directly affect the TyG index value, which is the primary variable of this study, and these people account for only about 13% of the total health check-up registry. Thus, we think this is acceptable in interpreting our main results. Second, due to the limitations of cross-sectional analyses, causality could not be established. Further prospective studies are needed to confirm our findings. Third, since we included a neurologically healthy population, the prevalence of SBI and the volume of WMH were relatively low. However, despite the small burdens, the association between the TyG index and cSVD was still prominent. This shows that there is a definite pathophysiological association between IR and cSVD. Last, since this study included participants that were visited over an eight-year periods from 2006 to 2013, changes in the diagnostic or experimental measurement methods may have occurred.

## Conclusion

In the current study, a high TyG index was associated with a higher prevalence and burden of cSVD in a neurologically healthy population. Most participants had so-called “normal ranges” of TG and glucose levels. Nevertheless, the TyG index showed a significant association with cSVD at this subclinical level. Thus, our findings indicate that the TyG index, a useful and straightforward indicator of one’s IR state, could be a marker for the prevalence and burden of cSVD encompassing SBI and WMH of different pathologies.

## Supplementary information

**Additional file 1: Table S1.** Baseline characteristics of the cohort (n = 2615).

## Data Availability

The datasets used and/or analyzed during the current study are available from the corresponding author on reasonable request.

## Abbreviations

IR: Insulin resistance
HOMA-IR: Homeostatic Model Assessment for Insulin Resistance
TG: Triglyceride
HDL: High-density lipoprotein
LDL: Low-density lipoprotein
TyG index: Triglyceride-glucose index
cSVD: Cerebral small vessel disease
WMH: White matter hyperintensity
SBI: Silent brain infarct
BMI: Body mass index
WBC: White blood cell
hs-CRP: High-sensitivity C-reactive protein
