# Supplementary material for: High triglyceride-glucose index is associated with subclinical cerebral small vessel disease in a healthy population: a cross-sectional study
Source: Cardiovasc Diabetol. 2020 May 6;19:53. doi: 10.1186/s12933-020-01031-6 (PMC7201807; doi:10.1186/s12933-020-01031-6)
Supplement: Supplementary file 1 — Additional file 1: Table S1. Baseline characteristics of the cohort (n = 2615). [file 12933_2020_1031_MOESM1_ESM.docx]

**Additional Files**

**Table S1. Baseline characteristics of the cohort (n = 2,615)**

| Age, y [SD] | 57 ± 8 |
| --- | --- |
| Sex, male, n (%) | 1,386 (53) |
| Body mass index, kg/m^2^ [SD] | 24.07 ± 3.04 |
| Use of antihypertensives, n (%) | 474 (18) |
| Use of antiplatelet agents, n (%) | 210 (8) |
| Systolic blood pressure, mmHg [SD] | 126 ± 16 |
| Diastolic blood pressure, mmHg [SD] | 76 ± 11 |
| Hemoglobin A1c, % [SD] | 5.8 ± 0.7 |
| Fasting glucose, mg/dL [SD] | 94 ± 19 |
| Insulin, µU/mL [SD]^*^ | 7.1 ± 4.7 |
| HOMA-IR, [SD]^*^ | 1.69 ± 1.30 |
| Total cholesterol, mg/dL [SD] | 202 ± 36 |
| LDL cholesterol, mg/dL [SD] | 129 ± 34 |
| HDL cholesterol, mg/dL [SD] | 55 ± 14 |
| Triglyceride, mg/dL [SD] | 117 ± 71 |
| TyG index [SD] | 8.47 ± 0.57 |
| White blood cell counts, x 10^3^/μL [SD] | 5.52 ± 1.66 |
| hs-CRP, mg/dL [SD] | 0.17 ± 0.65 |
| White matter hyperintensity volume, mL [SD] | 2.53 ± 5.78 |
| Silent brain infarct, n (%) | 209 (8) |

HOMA-IR = Homeostatic Model Assessment for Insulin Resistance, LDL = low-density lipoprotein, HDL = high-density lipoprotein, hs-CRP = high-sensitivity C-reactive protein, TyG index = triglyceride-glucose index

^*^These variables were measured in 2,051 participants
